# An Unusual Cause of End-Tidal Carbon Dioxide Rise During One-Lung Ventilation

**DOI:** 10.7759/cureus.41034

**Published:** 2023-06-27

**Authors:** Jason T Boyle, Andre F Gosling, Benjamin Wei, Abey S Abraham, Nishank Nooli

**Affiliations:** 1 Cardiothoracic Anesthesiology, University of Alabama at Birmingham School of Medicine, Birmingham, USA; 2 Cardiothoracic Surgery, University of Alabama at Birmingham School of Medicine, Birmingham, USA; 3 Cardiothoracic Anesthesiology, Cleveland Clinic Foundation, Cleveland, USA

**Keywords:** lung lobectomy, general thoracic surgery, hypercapnea, cardiothoracic anesthesia, capnothorax, end tidal carbon dioxide, one lung ventilation

## Abstract

A relatively common problem that may arise during one-lung ventilation is elevation of end-tidal carbon dioxide (ETCO2), which has several potential etiologies. This case report describes a 69-year-old woman with carcinoid tumor undergoing a robotic left lower lobectomy complicated by an acute rise in ETCO2 during one-lung ventilation, without an immediately identifiable cause. Thorough evaluation revealed CO2 leak through an open bronchial lumen resulting in an artificially high ETCO2 reading. This case report demonstrates the importance of performing a comprehensive assessment during acute changes in ETCO2 while also considering changes in the surgical field, which may contribute to these findings.

## Introduction

One-lung ventilation (OLV) is a technique for surgical field optimization, often utilized in thoracic surgery. While it can facilitate the achievement of good operating conditions, it is also associated with a myriad of complications, such as hypoxia [1], hypercarbia, acidosis, and hemodynamic instability [2]. Hypoxia is one of the most frequently encountered issues with OLV and there are several well-known strategies to mitigate it [1,3-4]. Dynamic hyperinflation may also occur during OLV, more frequently in patients with severe obstructive disease, potentially leading to hemodynamic instability and even cardiac arrest [2]. Although permissive hypercapnia has been shown to have beneficial effects, such as inhibiting systemic inflammation during OLV [5,6], acute extreme elevations of end-tidal carbon dioxide (ETCO2) can be detrimental and should be timely addressed. Our report focuses on an unusual cause of ETCO2 rise during OLV, resulting from a CO2 leak through an open bronchial lumen, which led to an artificially high ETCO2 reading. Written Health Information Portability and Accountability Act (HIPAA) authorization consent has been obtained for the publication of this case report.

## Case presentation

**Figure 1:**
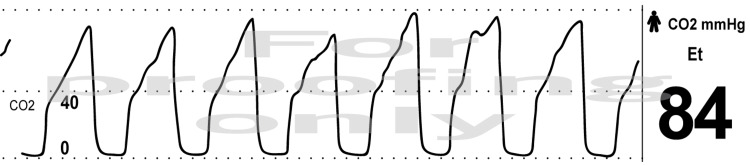
Acute Rise of End-Tidal Carbon Dioxide (ETCO2) - Intraoperative image showing the abnormal ETCO2 waveform seen during the case with steep upsloping morphology.

A 69-year-old woman (154cm, 165lbs, BMI 31) with a carcinoid tumor presented for robotic left lower lobectomy. General anesthesia was induced and a 35 Fr left-sided double-lumen tube (DLT) was subsequently inserted using flexible bronchoscopy for correct placement. Under direct visualization both the tracheal and bronchial cuffs were inflated with a good seal. The bronchial lumen was clamped, and the left lung was allowed to collapse. This was followed by CO2 insufflation of the left hemithorax to a pressure of 10 mmHg. There was optimal left lung isolation and no issues with ventilating the right lung. As the patient did not have significant cardiac or pulmonary disease, it is a common institutional practice to not place an arterial line. When the sleeve resection and reconstruction was being performed, the ETCO2 acutely rose from the mid-30s mmHg to the mid-80s mmHg with an abnormal capnography waveform affecting phase 3 without changes in the flow volume loop (Figure 1).

**Figure 2:**
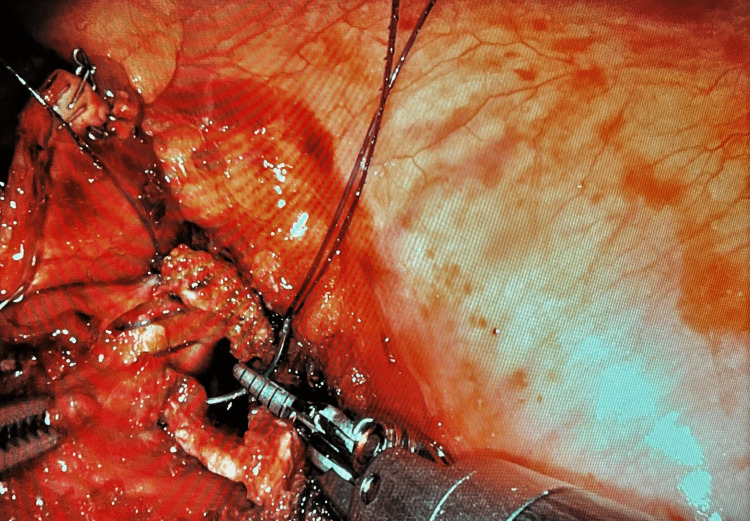
Surgically Transected Left Lower Lobe Bronchus - Intraoperative image of the surgical field during the period of elevated ETCO2, showing an opened airway segment being actively sutured.

Despite this significant change, the patient’s vital signs and ventilatory parameters like peak airway pressure, mean airway pressure and minute ventilation remained stable. A thorough systematic evaluation of the patient and anesthesia monitors was performed to rule out potential causes including fever, malignant hyperthermia, exhaustion of CO2 absorber, expiratory valve malfunction, subcutaneous emphysema, CO2 embolism and it failed to reveal a cause for the acute rise. It was then noticed that the surgical team was working on the transected left lower lobe bronchus, which was open in the surgical field (Figure 2).

**Figure 3:**
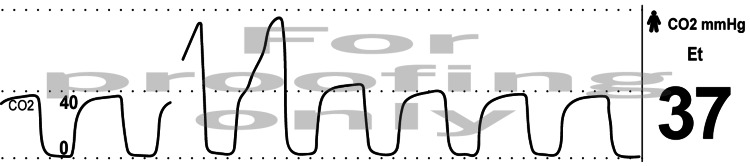
Acute Resolution of Elevated ETCO2 - Intraoperative image of the acute transition from the abnormal ETCO2 waveform morphology to baseline after addition of air into the bronchial cuff of the double-lumen endotracheal tube resolving the CO2 leak.

At this point it was hypothesized that it could be spurious ETCO2 elevation due to leakage of capnothorax around the previously inflated bronchial cuff, thus, additional air was added to the bronchial cuff of the DLT, which resulted in the immediate return of the ETCO2 tracing to prior levels and morphology (Figure 3).

This proved the hypothesis that the acute rise of ETCO2 was a consequence of insufflation CO2 leak into the open airway segment with enough pressure to seep around the bronchial cuff of the DLT during expiration resulting in elevated ETCO2 reading, unrelated to the patient’s physiology. As the patient remained hemodynamically stable and there were no further issues with ventilation during the procedure, we did not consider escalating care to place an arterial line or send an arterial blood gas. The patient was extubated uneventfully at the end of the procedure. Pathology confirmed it to be a typical carcinoid tumor, 6.1 cm in size and T3N0 Stage IIB. No post operative adjuvant therapy was given. The patient had a surveillance bronchoscopy after one year and computerized tomography scan at sixth month, one year, and two-year follow-ups showing no evidence of tumor recurrence.

**Figure 4:**
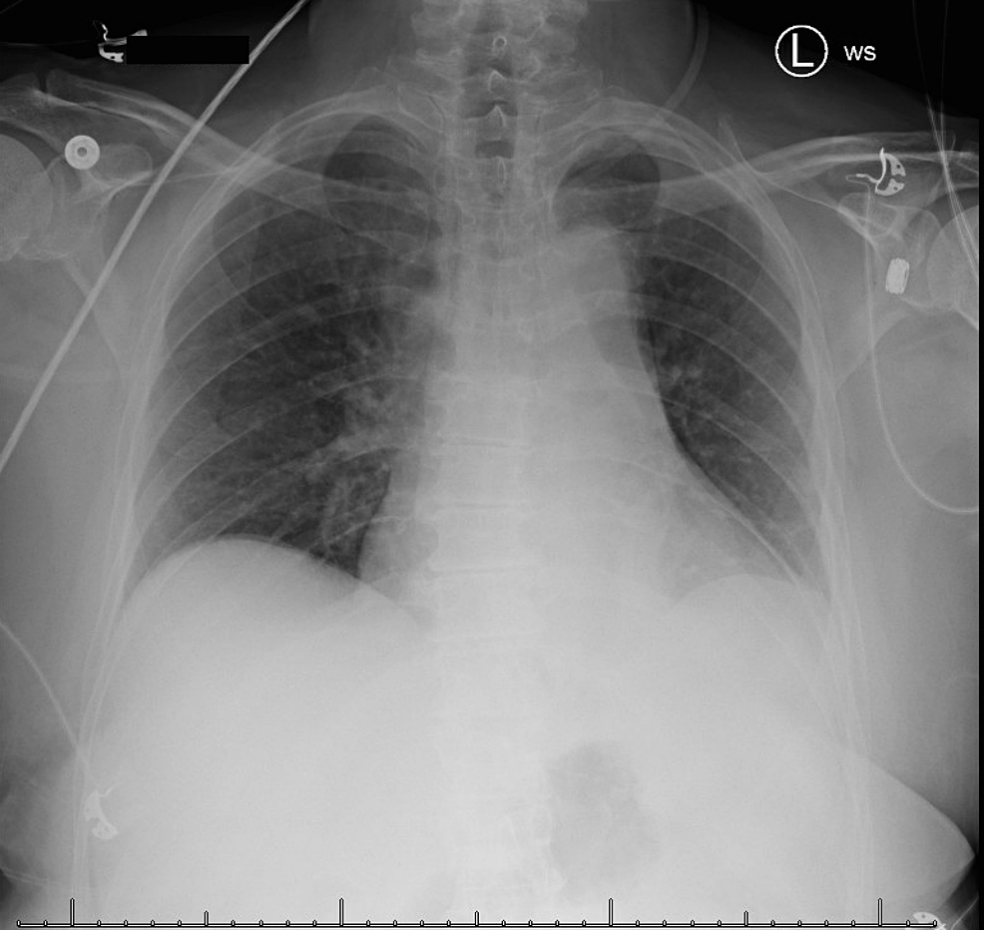
Pre-operative Chest X-ray. Chest X-ray demonstrating partial atelectasis of the left lower lobe secondary to the tumor. No pneumothorax or pleural effusion.

The patient’s pre-operative chest X-ray revealed partial atelectasis of the left lower lobe secondary to the tumor (Figure 4).

## Discussion

Elevated ETCO2 is not an uncommon finding under general anesthesia. Causes of increased ETCO2 include decreased CO2 elimination [7], which can be seen with inadequate ventilation and malfunction of anesthesia equipment [8]; increased production of CO2 from hypermetabolic states [9]; and iatrogenic causes such as CO2 insufflation. After ruling out the most obvious causes, monitor malfunction or spurious readings should be considered [10]. It is important to have a systematic approach to the investigation of potential etiologies.

In our case, the evaluation started with the patient. The patient was afebrile, normotensive, and in normal sinus rhythm. Therefore, a hypermetabolic state was extremely unlikely. Minute ventilation, peak airway pressures, and O2 saturation remained unchanged, suggesting no issues with ventilation. The ETCO2 tracing returned to baseline during inspiration, ruling out increased inspired CO2 as a cause. CO2 embolism was contemplated due to the acute nature of the event, but there was no change in hemodynamics. Furthermore, we did not observe any crepitus on palpation of the chest and there was no abdominal wall injury per surgical team, thus excluding subcutaneous emphysema. After a thorough evaluation of the patient and anesthesia machine proved negative, monitor malfunction or spurious reading was considered. It was noted that there was an open airway segment in the surgical field, which was thought to be the culprit. When additional air was added to the bronchial cuff of the DLT, the ETCO2 tracing immediately returned to prior levels and morphology.

Reconstruction of an airway for a bronchoplasty or sleeve resection can take about 20 to 45 minutes, under typical conditions; therefore, managing a rise in ETCO2 due to the aforementioned cause will likely require an intervention. In this case, addressing a small leak around the bronchial cuff was sufficient to mitigate the increased ETCO2. Decreasing or eliminating CO2 insufflation into the surgical field can also help; this can often be done without negative effect on the surgeon’s view while the reconstruction is being performed if the operative lobe has already been removed. Some of the complications encountered during OLV are depicted in Table 1.

**Table 1:** Hypercapnea causes and management strategy during one-lung ventilation

PaCO2-ETCO2 gradient	Causes
Normal	Hypoventilation (cranial disorder, myasthenia gravis, central sleep apnea, drug toxicity, exogenous CO2 exposure during insufflation)
Increased	Pulmonary embolism, hypovolemic shock, high positive end-expiratory pressure, pulmonary edema, chronic obstructive pulmonary disease
Management	Permissive hypercapnia is acceptable, unless the patient has contraindications (e.g., intracranial hypertension, seizure disorder, pulmonary hypertension and right ventricular dysfunction); increase minute-ventilation to augment CO_2 _elimination

Similar cases of CO2 leak into the airway from the surgical field resulting in spuriously elevated ETCO2 have been previously reported [11,12], and Ravi et al. suggested utilizing decreased insufflation pressure and increased positive end-expiratory pressure (PEEP) to counteract the capnothorax leakage [12]. However, we believe that adding air to the bronchial cuff is a more effective way of eliminating the communication and correcting the leakage.

## Conclusions

This case demonstrates the importance of performing a comprehensive assessment in the course of acute changes in ETCO2 during OLV. We highlight the initial patient assessment based on our monitor readings, followed by checking the ventilatory parameters and ensuring no defect with the anesthesia machine. If the hypercapnea persists with unknown etiology, it is important to communicate with the surgeon, given the possibility of a surgical cause, as demonstrated in our case.
